# Allergen Component Testing: Key in Diagnosing Atypical Pollen-Food Allergy Syndrome

**DOI:** 10.7759/cureus.58722

**Published:** 2024-04-22

**Authors:** Melvin Lee Qiyu, Tom Dawson, Phoebe Moulsdale

**Affiliations:** 1 Pediatrics, Worcestershire Acute Hospitals NHS Trust, Worcester, GBR

**Keywords:** pollen-food allergy syndrome, food allergies, allergen component testing, knowledge of allergic rhinitis, pollen allergies

## Abstract

This case report details the complex presentation, diagnosis, and management of a teenager with pollen-food allergen syndrome (PFAS), formerly known as oral allergy syndrome. PFAS, mediated by immunoglobulin E (IgE) antibodies, stems from the cross-reactivity between pollens and uncooked plant-based foods, leading to a spectrum of symptoms, such as itching or tingling of the oral cavity. A UK survey indicated an average PFAS prevalence of 2%, with apples, hazelnuts, and kiwifruit commonly implicated. The presented case involved a 15-year-old girl referred from the respiratory clinic to the allergy clinic due to episodes of sore throat and urticaria rash following Nutella (chocolate paste containing hazelnut) and peanut consumption. Extensive diagnostic measures, including specific IgE testing, skin prick tests, and allergen component testing, revealed cross-reactivity between Bet v 1 and hazelnut allergens. The patient's atopic history, encompassing poorly controlled asthma, allergic rhinitis, and eczema, added layers of complexity to the diagnosis. Management strategies comprised dietary advice, allergen avoidance, and potential consideration of aeroallergen immunotherapy. A comprehensive dietary plan emphasized abstaining from specific foods and raising awareness of potential reactions. The patient, following guidance from the allergy clinic, exhibited improvements in allergic rhinitis and oral symptoms. This case underscores the importance of allergen component testing in diagnosing atypical PFAS presentations and tailoring management plans. Ongoing collaboration between healthcare providers, detailed patient education, and regular follow-ups are crucial for effective PFAS management and long-term care.

## Introduction

Pollen-food allergen syndrome (PFAS), formerly referred to as "oral allergy syndrome," involves an immune response mediated by immunoglobulin E (IgE) antibodies [1]. It arises due to the cross-reactivity between pollens and uncooked plant-based foods, such as vegetables, fruits, tree nuts, and others. The primary symptoms of PFAS often include oral irritation and a sensation of tightness in the throat. In a subset of these individuals, additional symptoms like hives, asthma, or potentially life-threatening anaphylaxis can manifest subsequent to the initial oral discomfort [2].

The prevalence of PFAS is recognized to fluctuate based on the geographic location and aeroallergens prevalent in that area [2]. According to a UK survey involving 3,590 participants, the average prevalence of PFAS was 2% (0.8-4.1%), with apples, hazelnuts, and kiwifruit being reported as the common sources. Most individuals in this study experiencing PFAS symptoms reported their first encounters with these symptoms before the age of 20 [3]. The study may have sampling bias from self-reported data and location bias with varying PFAS rates between regions, especially with a higher response rate from urban [3]. The emergence of PFAS is linked to sensitization through aeroallergens, resulting in a subsequent cross-reactivity between pollens and similar epitopes found in plant-derived foods. PFAS is associated with class II food allergens, which exhibit a relatively unstable profile compared to class I food allergens. Class II food allergens are aero-allergens that cause sensitization occurring through the respiratory tract, such as primary birch pollen allergen Bet v 1. Immune responses to these allergens can cross-react with related food allergens, such as the major apple allergen Mal d 1 [4]. These class II allergens tend to lose their allergenic properties when exposed to gastric acid, heat, and digestive enzymes, which explains the common limited presentation in oral symptoms [5]. 

Allergen component testing is a specialized type of allergy testing that identifies individual proteins or components of allergens rather than the whole allergen. For example, instead of testing for the whole peanut allergen, it might identify specific peanut proteins like Ara h 1 and Ara h 2 [6]. It helps differentiate between primary sensitization and cross-reactivity-induced sensitization.

## Case presentation

A 15-year-old girl was previously referred from the respiratory clinic to the allergy clinic due to episodes of sore throat and urticaria rash following consumption of Nutella and peanuts. All nut avoidance was advised prior to admission to the allergy clinic. Specific IgE for nuts was conducted by the respiratory team. The results of the initial IgE test were as follows: sIgE for hazelnut, 9.32 kU/L; mixed nuts (peanut, hazelnut, Brazil nut, almond and coconut), 11.8 kU/L; peanuts, 1.9 kU/L; Brazil nut, 0.01 kU/L; almond, 0.9 kU/L; coconut, 0.08 kU/L; and total IgE level, 473 kU/L. A result ≥0.35 kU/L indicates a positive result (Table 1).

**Table 1:** Summary of skin prick tests (SPTs) and specific immunoglobulin E (IgE) levels. A positive skin prick test (SPT) is indicated by a value of 3 mm and above. A positive specific immunoglobulin E (IgE) level is indicated by a value of 0.35 kU/L and above.

Aeroallergen SPT	Result	Food SPT	Result	Specific IgE	Result (kU/L)
Mixed trees 1 (silver birch, alder, hazelnut)	7 mm	Peanut	0mm	Mixed nut	11.8
Mixed trees 2 (white poplar, elm, white willow)	2 mm	Almond	1mm	Hazelnut	9.32
Mixed trees 3 (Euro beech, oak, ash)	4 mm	Cashew	0mm	Peanut	1.90
Silver birch	8 mm	Hazelnut	4mm	Brazil	0.01
Six grass mix	4 mm			Almond	0.90
Timothy grass	4 mm			Coconut	0.08
House dust mite *Dermatophagoides farinae*	1 mm			Silver birch	29.80
House dust mite *Dermatophagoides* *pterinissus*	2 mm			Timothy grass	20.5
Alternaria alternata	2 mm			Total IgE	473
Aspergillus fumigatus	0 mm				
Cladosporium	1 mm				

Upon presentation to the allergy clinic, a detailed history and examination were taken. The patient reported episodes of oral symptoms, such as throat pruritus and throat tightness, following immediate consumption of hazelnut spread (less than five minutes). Upon consumption of roasted peanuts, her only symptom was a painful throat without other symptoms, such as urticarial rash, vomiting, diarrhea, or airway difficulties. In addition, she stated that following raw apple and carrot consumption, she experienced a tingling sensation around her lips but was asymptomatic on cooked carrot and apple crumble. Bananas, raisins, and pears all also gave her a tingling sensation on the lips with throat pain. These had ultimately led to avoidance of the abovementioned food from her diet in the past. Interestingly, she also mentioned unspecific, intermittent episodes of urticarial rash and peripheral angioedema of hands throughout the years without any specific triggers. On examination, the patient had good air entry bilaterally with no added sounds. The nasal cavity exhibited inflammation, accompanied by intermittent sniffling. There were also patches of dry skin noted over both antecubital fossa areas. Apart from these findings, the rest of the examination yielded normal results.

She had a significant atopy history, including eczema, allergic rhinitis, and asthma. Her poorly controlled asthma was managed by a local pediatric respiratory team with regular medications, including fluticasone furoate/vilanterol trifenatate 99 mcg/22 mcg one puff per day, and salbutamol 100 mcg can be used up to 10 puffs if required with aerochamber, montelukast 4 mg nocte, and theophylline 300 mcg BD. Her eczema is usually well-controlled, and she is currently on regular emollient and topical 0.5% hydrocortisone cream if required. She suffered significant allergic rhinitis all year round and has been on over-the-counter antihistamine fexofenadine 30 mg OD for relief of symptoms. 

Several allergens for the skin prick test (SPT) were decided based on the history, including peanuts, almonds, cashew, hazelnut, house dust mites (*Dermatophagoides farina* and *Dermatophagoides pteronyssinus*), six grass mixes, timothy grass, silver birch, *Alternaria alternata*, *Aspergillus fumigata,* and *Cladosporium*. Positive SPT results included hazelnut (4 mm), six grass mixes (4 mm), timothy grass (4 mm), and silver birch (8 mm) (Table 1).

Due to the atypical presentation with oral and skin symptoms, an allergen component test was carried out with additional specific IgE testing on Timothy grass and the C3/C4 level due to angioedema. Positive results from component testing included peanut Ara H 8-9.87 kU/L, birch Bet V 1-31.7 kU/L, and hazelnut Cor A 1-21.3 kU/L (Table 2). Based on the results of the component testing, PFAS was confirmed, and both primary nut allergy and LTP allergy were excluded. The C3 and C4 levels were assessed, which showed a C3 level of 1.66 g/L and a C4 level of 0.49 g/L. These normal results also ruled out hereditary angioedema. 

**Table 2:** Summary of the allergen component testing results. A positive result is indicated by a level of 0.35 kU/L and above.

Component	Results (kU/L)
Peanut Ara h 1	0.02
Peanut Ara h 2	0.01
Peanut Ara h 3	0.02
Peanut Ara h 8	9.87
Birch Bet v 1 PR-10	31.70
Birch Bet v 2	0.02
Hazelnut Cor a 1	21.30
Hazelnut Cor a 8	0.19
Hazelnut Cor a 9	0.01

The patient was then discharged from the allergy clinic at that point following a detailed explanation of the disease, dietary advice, and discussion regarding potential aeroallergen immunotherapy if her asthma is better controlled in the future. Comprehensive dietary recommendations involve abstaining from foods containing peanuts and hazelnuts and avoiding raw fruits that trigger oral irritability while permitting their consumption in cooked forms. The advice includes awareness of the potential for both local and systemic reactions, although no adrenaline auto-injector was prescribed at this point due to having no previous episode of anaphylaxis. Further consideration of adrenaline prescription will be made if systemic reactions will be experienced in the future. She was also started on intranasal budesonide 128 microgram one spray twice a day for better control of her allergic rhinitis.

After a subsequent follow-up with the respiratory team three months later, the patient demonstrated improved control over her asthma. However, the mother expressed reluctance to reduce the theophylline dosage. As a result, arrangements were made for a follow-up appointment in the respiratory clinic for a thorough review in six months. Notably, the patient's allergic rhinitis and oral symptoms had shown improvement following the guidance provided in the allergy clinic.

## Discussion

As per a survey conducted in the United States, the median progression rate of PFAS to systemic symptoms is approximately 5%, ranging from 1% to 28%. A variety of foods, such as tree nuts, apples, bananas, and carrots, were identified as common triggers for systemic reactions [7]. Atypical systemic symptoms in PFAS include asthma, angioedema, urticaria, and anaphylaxis [8].

Diagnosing the mentioned case can be particularly challenging without allergen component testing, as specific IgE levels and skin prick tests alone do not offer insights into cross-reactivities. Among children sensitized to Bet v 1, the predominant allergens causing cross-reactivity are alder Aln g 1 (96.9%), hazelnut Cor a 1.0401 (87.6%), and apple Mal d 1 (87.6%) [9]. Cor a 1 is a PR-10 protein that shared a significant cross-reactivity with Bet v 1. Patients with a high level of specific IgE to hazelnuts without sensitization to storage proteins typically can tolerate hazelnuts or may experience only mild oropharyngeal symptoms [10]. The interaction of IgE with Ara h 8 was impeded by Bet v 1, as evidenced in both peanut extract immunoblotting and radioallergosorbent test (RAST) inhibition. This observation indicates a cross-reaction between Bet v 1 and the homologous peanut allergen Ara h 8 [11].

As per the guidelines provided by the British Society of Allergy and Clinical Immunology (BSACI), the primary approach for managing PFAS involves avoiding known triggers. While there is limited evidence regarding the effectiveness of immunotherapy for pollens, there are some positive outcomes reported for oral or sublingual immunotherapy targeting foods [12]. An observational study conducted in Germany found an improvement in the overall severity score of PFAS from 94.9% to 36.9% following pollen-specific sublingual immunotherapy [13]. However, further studies are needed to better understand and establish the efficacy of this approach.

## Conclusions

The presented case involves a teenager with PFAS, characterized by oral and atypical skin symptoms following the consumption of hazelnut spread, peanuts, and various raw fruits. Comprehensive diagnostic approaches, including specific IgE testing, skin prick tests, and allergen component testing, were employed to discern the specific allergens responsible for the symptoms. The patient exhibited cross-reactivity between Bet v 1 and hazelnut allergens, highlighting the complexity of PFAS.

The patient's atopic history, including poorly controlled asthma, allergic rhinitis, and eczema, added complexity to the case. Regular follow-ups and collaboration between allergy and respiratory teams were essential for monitoring and optimizing asthma control. The case underscores the significance of allergen component testing in diagnosing atypical presentations of PFAS and tailoring management plans accordingly. The improvement in symptoms, especially in allergic rhinitis and oral manifestations, following guidance from the allergy clinic highlights the importance of targeted interventions in managing PFAS.
